# Dynamics of chiral solitons driven by polarized currents in monoaxial helimagnets

**DOI:** 10.1038/s41598-020-76903-8

**Published:** 2020-11-24

**Authors:** Victor Laliena, Sebastian Bustingorry, Javier Campo

**Affiliations:** 1 Aragon Nanoscience and Materials Institute (CSIC-University of Zaragoza) and Condensed Matter Physics Department, University of Zaragoza, C/Pedro Cerbuna 12, 50009 Zaragoza, Spain; 2 Instituto de Nanociencia y Nanotecnología, CNEA-CONICET, Centro Atómico Bariloche, R8402AGP Bariloche, Río Negro Argentina

**Keywords:** Magnetic properties and materials, Spintronics

## Abstract

Chiral solitons are one dimensional localized magnetic structures that are metastable in some ferromagnetic systems with Dzyaloshinskii–Moriya interactions and/or uniaxial magnetic anisotropy. Though topological textures in general provide a very interesting playground for new spintronics phenomena, how to properly create and control single chiral solitons is still unclear. We show here that chiral solitons in monoaxial helimagnets, characterized by a uniaxial Dzyaloshinskii–Moriya interaction, can be stabilized with external magnetic fields. Once created, the soliton moves steadily in response to a polarized electric current, provided the induced spin-transfer torque has a dissipative (nonadiabatic) component. The structure of the soliton depends on the applied current density in such a way that steady motion exists only if the applied current density is lower than a critical value, beyond which the soliton is no longer stable.

Magnetic structures of nanometric size, like domain walls, vortices, or skyrmions, attracted great attention since they are very promising as the building blocks of spintronic components such as memories, logical gates, etc. To be useful, they have to satisfy at least two essential requirements: (1) be (meta)stable, and (2) move in a controlled way under the action of external stimuli, such as applied magnetic fields or electric currents. Chiral solitons, which are one dimensional solitonic magnetic structures of topological nature, have received comparatively much less attention, although they are also potentially useful in spintronics and digitalization applications. In monoaxial helimagnets, the Dzyaloshinskii–Moriya interaction (DMI) and the uniaxial magnetic anisotropy (UMA) are the key ingredients that provide the chiral soliton metastability, and, at low enough temperatures and applied magnetic field chiral solitons condense and form a stable chiral soliton lattice (CSL)^1–4^. Skyrmions condense also in some regions of the phase diagram of cubic helimagnets, in the form of skyrmion lattices^5–7^.

In monoaxial helimagnets the DMI exists only along one crystallographic axis, the DMI axis. These materials are strongly anisotropic and the magnetic structures that host depend on the direction of the applied magnetic field with respect to the DMI axis. At low temperature and low field the magnetic equilibrium state is spatially modulated: a CSL if the field is applied perpendicular to the DMI axis and a conical helix if it is parallel^1,2^. If the field direction is neither perpendicular nor parallel, a modulated structure appears connecting smoothly the two limiting cases^8^. The phase diagram and the nature of the phase boundaries were theoretically studied in the case of perpendicular field^9,10^, and in the case of magnetic fields applied in arbitrary directions^11–14^. Among the known monoaxial helimagnets [${\text {CrNb}}_3{\text {S}}_6$, CrTa$_3$S$_6$, CuB$_2$O$_4$, CuCsCl$_3$, Yb(Ni$_{1-x}$Cu$_x$)$_3$Al$_9$, Ba$_2$CuGe$_2$O$_7$]^15–21^, the most studied is ${\text {CrNb}}_3{\text {S}}_6$, which may be considered the archetypal monoaxial helimagnet. Its phase diagram and the nature of the phase boundaries, determined experimentally by several groups^22–26^, agree well with the theoretical predictions. The dynamics of the CSL and of the conical helix has been studied theoretically in some regimes^27–30^ and experimentally^31–33^ by several groups. The dynamics of isolated chiral solitons in monoaxial helimagnets, however, has not been addressed either theoretically or experimentally, although a solitonic structure propagating over a CSL background has been theoretically constructed via the Bäcklund transformation^34^. It has been also pointed out that the properties of isolated solitons play a prominent role in determining the nature of the transition to the CSL phase^12,13^.

The dynamics of one dimensional magnetic solitons is being actively investigated in recent years, both experimentally and theoretically. But all this investigations concern domain walls^35–42^ and 360$^{\text {o}}$ domain walls^43–45^ in non chiral materials or in heterostructures with interfacial DMI. Chiral solitons in monoaxial helimagnets, however, have not been considered in spite that, as a new route to spintronic devices, they may have advantages over skyrmions, whose motion is gyrotropic and therefore difficult to control^46–48^, and over domain walls, since chiral solitons may provide a different mechanism to avoid pinning effects hindering domain wall motion^38,39,42^. As we will show here, chiral solitons in monoaxial helimagnets move steadily under the application of a polarized current, reaching velocities of the order of $100\,{\text {m/s}}$ for currents around $100\,{\text {GA/m}}^2$. Furthermore, if the current is large enough the stability of the soliton is compromised and the system is forced to a homogeneous magnetization state.

The soliton can be controlled and manipulated only if it is metastable. Otherwise, any external stimulus will destroy it. Therefore, in the present work we first determine the domain of metastability of chiral solitons in monoaxial helimagnets as a function of the UMA and the magnetic field strength applied perpendicularly to the DMI axis. Then we study the response of the metastable soliton to the spin transfer torque delivered by a polarized electric current. We found (1) that for the values of UMA typical of these systems the soliton is metastable in a broad range of the magnetic field strength above the critical field; (2) that the soliton reaches a steady motion regime with a mobility proportional to the current density provided that the spin transfer torque has a non-adiabatic component and the current density is below a critical value; and (3) that current densities above the critical value destroy the soliton, so that there is no analogous to Walker precessional regime characteristic of domain walls.

## Statics of isolated chiral solitons in monoaxial helimagnets

**Figure 1:**
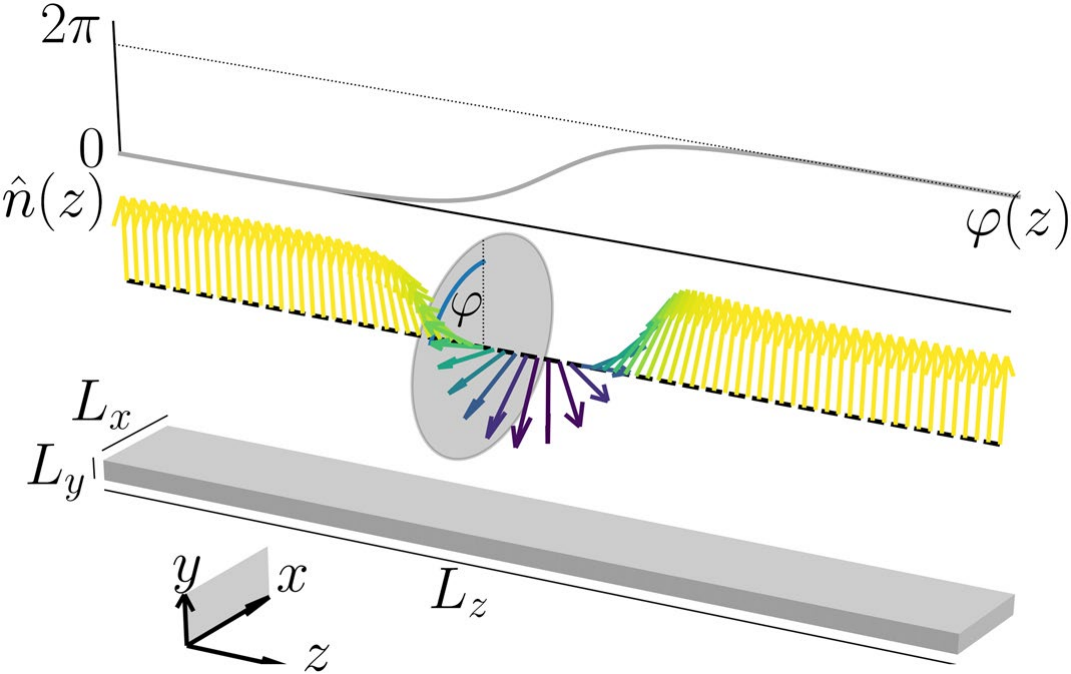
Structure of the quiral soliton and geometry of the system. The $\chi = +1$ chiral soliton is described by $\hat{n}(z)$ with $\varphi (z)$ given by $\varphi _0$ in Eq. (5) (top). The polar angle is $\theta = \pi /2$ and thus the normalized magnetization $\hat{n}$ is in the *x*-*y* plane and rotates along the chiral axis, as indicated. The dimensions of the modeled magnetic track is schematically shown in the bottom figure.

Consider a magnetic nanometer size track with dimensions $L_y\ll L_x \ll L_z$ (see Fig. 1), made of a monoaxial helimagnet, such as ${\text {CrNb}}_3{\text {S}}_6$, with chiral axis along $\hat{z}$. Its magnetic energy is given by $E = \int d^3x W$, with

1 $$W = A\sum\limits_{{i = x,y,z}} {\partial _{i} } \hat{n} \cdot \partial _{i} \hat{n} - D\hat{z} \cdot (\hat{n} \times \partial _{z} \hat{n}) - K(\hat{z} \cdot \hat{n})^{2} - M_{{\text{S}}} \vec {{B}} \cdot \hat{n},$$

where $\hat{n}$ is a unit vector field that describes the magnetization direction at each point of the film, *A*, *D*, and *K* stand for the exchange stiffnes constant, and the DMI and UMA strength constants, respectively, $M_{\text {S}}$ is the saturation magnetization, and ${\vec{B}}$ is the applied magnetic field. The DMI acts only along the $\hat{z}$ axis, defining thus a monoaxial helimagnet, and it is of bulk type and not interfacial, in spite that the track lies in a thin film in the $y=0$ plane. The sign of *D* is reversed if we reverse the direction of the $\hat{z}$ axis, so that, with no loss of generality, we take $D>0$. It is also convenient to introduce $q_0=D/2A$, which has the dimensions of inverse length, and the dimensionless parameters $\kappa =4AK/D^2$ and ${\vec{h}}=(2AM_{\text {S}}/D^2) {\vec{B}}$. For the sake of simplicity, we ignore the magnetostatic energy, whose effect in an infinite system in which the magnetization depends only on *z* is completely absorbed in the UMA^49^. In a finite system, the boundary conditions introduce a dependence of the magnetization in *x* and *y* and the surface magnetic poles induce a dipolar field. The results presented here should be valid for system sizes $L_x$ and $L_y$ much larger than $A/K_S$, with $K_S$ the surface anisotropy, and than $\ell _{{\text {ex}}}=\sqrt{2A/\mu _0M_S^2}$, so that magnetostatic effects at the surface of the system can be ignored. Typical values of $A/K_S$ and $\ell _{{\text {ex}}}$ are in the scale of 10 nm, and then our results should be of relevance for systems with $L_x$ and $L_y$ larger than several tens nm.

The dynamics obeys the Landau–Lifschitz–Gilbert (LLG) equation

2 $$\begin{aligned} \partial _t\hat{n}= \gamma {\vec{B}} _{{\text {eff}}}\times \hat{n}+ \alpha \hat{n}\times \partial _t\hat{n}+{\vec{\tau} }, \end{aligned}$$

where $\alpha$ and $\gamma$ are the Gilbert damping parameter and the gyromagnetic constant, respectively, and ${\vec{\tau} }$ stands for some applied torque not included in the energy (1). The effective field acting on $\hat{n}$ is given by

3 $$\begin{aligned} {\vec{B}}_{{\text {eff}}} = \frac{2A}{M_{\text {S}}} \!\left[ \nabla ^2\hat{n}- 2q_0\hat{z}\times \partial _z\hat{n}+ q_0^2\kappa (\hat{z}\cdot \hat{n})\hat{z}+ {q_0^2}\vec {h} \right] . \end{aligned}$$

The Euler–Lagrange equations describing the static solutions are ${\vec{B}}_{{\text {eff}}} = \lambda \hat{n}$, where $\lambda$ is a Lagrange multiplier enforcing the constraint $\hat{n}^2=1$. If the applied field is perpendicular to the chiral axis, a single chiral soliton is a metastable static solution. Taking $\vec {h} = h_y{\hat{y}}$ and using the parametrization

4 $$\begin{aligned} \hat{n}= -\sin \theta \sin \varphi \hat{x}+ \sin \theta \cos \varphi \hat{y}+ \cos \theta \hat{z}, \end{aligned}$$

this well known solution is obtained by seeking for a solution of the Euler–Lagrange equations with depends only on *z* and which has a constant $\theta$. One obtains $\theta =\pi /2$ and the Sine–Gordon equation, $d^2\varphi /dz^2=q_0^2h_y\sin \varphi$. The solutions $\varphi _0$, that obey the boundary conditions (BCs) $\varphi _0(z=-\infty )=0$ and $\varphi _0(z=+\infty ) = \chi 2\pi$, with helicities $\chi =\pm 1$, are chiral solitons given by

5 $$\begin{aligned} \varphi _0(z) = 4 \chi \arctan {\text {e}}^{z/\Delta _0}. \end{aligned}$$

In contrast with domain walls, the soliton width, $\Delta _0=1/(q_0\sqrt{h_y}) = \sqrt{2A/(M_{\text {S}}B_y)}$, is independent of *K* and *D* and it is controlled by the applied magnetic field. This is a particularly interesting feature of chiral solitons. The generic shape of the soliton with helicity $\chi =+1$ is shown in Fig. 1.

The soliton exists as a stationary point of the energy even in a simple ferromagnet. Indeeed, the DMI and the UMA do not enter the Sine–Gordon equation, but, as we will see in this section, they are crucial to provide metastability to the chiral soliton. The soliton adds to the ferromagnetic (FM) state energy, $E_{{\text {FM}}}$, a contribution

6 $$\begin{aligned} \Delta E_{\text {S}} = L_xL_y2Aq_0\left( 8\sqrt{h_y}-2\pi \chi \right) . \end{aligned}$$

The term proportional to $\chi$ comes from the DMI, so that in absence of DMI the soliton is at most metastable $(\Delta E_{\text {S}}>0)$, and the solitons of both helicities are degenerated. The DMI lifts the degeneracy, lowering the energy if $\chi =+1$ and raising it otherwise. Below the critical field $h_{yc}=\pi ^2/16$, the energy of the favored soliton becomes negative, and the proliferation of solitons with the proper helicity ($\chi =+1$) is energetically favorable. Therefore, they condense forming a CSL^1,2,50,51^. Since the FM state remains metastable below the critical field, it makes sense to study isolated chiral solitons for $h_y<h_{yc}$.

To analyze the metastability of a single soliton, let us write the magnetic configuration $\hat{n}$ as

7 $$\begin{aligned} \hat{n}= (1-\xi _t^2-\xi _z^2)^{1/2}\hat{n}_0 + \xi _t\hat{z}\times \hat{n}_0 + \xi _z\hat{z}, \end{aligned}$$

where $\hat{n}_0$ stands for the soliton configuration and $\xi _t$ and $\xi _z$ are two real fields that describe the fluctuations around $\hat{n}_0$. Expanding the energy in powers of $\xi$ up to second order we get

8 $$\begin{aligned} E = E_{{\text {FM}}}+\Delta E_{\text {S}} + 2A\int d^3x \big (\xi _t K_t \xi _t + \xi _z K_z \xi _z\big ) + O(\xi ^3), \end{aligned}$$

where $K_t$ and $K_z$ are the differential operators

9 $$\begin{aligned} K_t= & {} -\nabla ^2 - \frac{1}{2}\varphi _0^{\prime \,2} + q_0^2h_y, \end{aligned}$$

10 $$\begin{aligned} K_z= & {} -\nabla ^2 - \frac{3}{2}\varphi _0^{\prime \,2} + q_0\varphi _0^\prime + q_0^2(h_y-\kappa ),\quad \end{aligned}$$

The terms linear in $\xi$ are absent in Eq. (8) since the soliton is a stationary point of the energy. The soliton is metastable if $K_t$ and $K_z$ are both positive (semi)definite. The time evolution of small excitations around the static configuration $\hat{n}_0$ is governed by the following two equations, which can be derived from the LLG equation (2):

11 $$\begin{aligned} \partial _t \xi _t = \frac{v_0}{q_0}\big (-K_z\xi _z-\alpha K_t\xi _t\big ), \qquad \partial _t \xi _z = \frac{v_0}{q_0}\big (K_t\xi _t-\alpha K_z\xi _z), \end{aligned}$$

where $v_0 = 2\gamma Aq_0/M_{\text {s}}$. Since the energy always decreases with time due to the damping term in the LLG equation, $\xi _t$ and $\xi _z$ will tend to zero as $t\rightarrow \infty$ if $\hat{n}_0$ is a local minimum of the energy and no additional external torque is applied, and therefore the soliton is metastable.

The spectrum of $K_z$ and $K_t$ is studied in detail in the Supplementary Information S1. Since $K_t$ is a Schrödinger operator corresponding to a Pöschl–Teller potential, exactly solvable, it is easy to verify that it is always positive semidefinite. Hence, the soliton stability is determined by the lowest lying eigenvalue of $K_z$. Without DMI, $K_z$ corresponds also to a Pöschl–Teller potential and it is easy to verify that the solitons of both chiralities are metastable if $h_y<-\kappa /3$. Notice that the DMI is removed by taking the limit $q_0\rightarrow 0$, keeping nonzero $q_0^2h_y$ and $q_0^2\kappa$. It amounts just to remove the term $q_0\varphi _0^\prime$ in Eq. (10). In the presence of DMI the spectrum of $K_z$ cannot be analytically determined and we have to resort on numerical methods.

**Figure 2:**
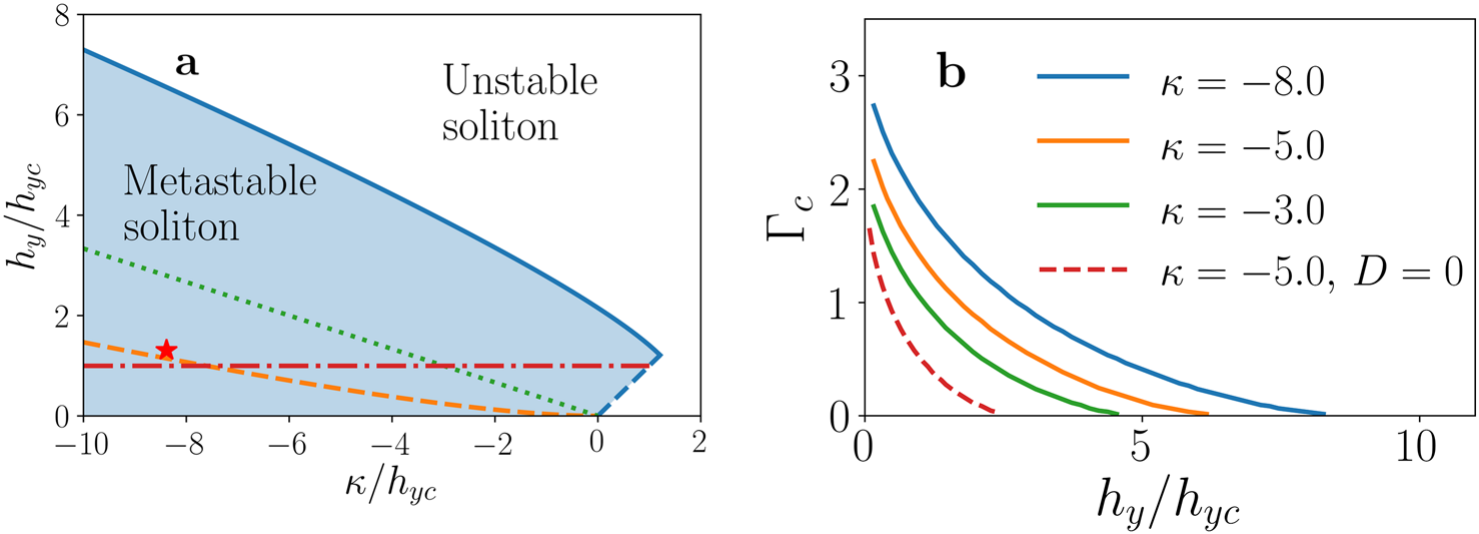
Limits of stability of the chiral soliton. (**a**) Stability diagram of the chiral soliton for $D>0$, as a function of anisotropy and applied field. The blue continuous line corresponds to the stability limit for $\chi = +1$, with the dashed blue line indicating the onset of instability of the whole system against tilting towards the $\hat{z}$ direction. The orange dashed line corresponds to the stability limit for $\chi = -1$ case. The green dotted line is the stability limit for $D = 0$ and $\chi = \pm 1$. Below the red dash-dotted line the FM state is itself metastable, the ground state being a CSL. The red star indicates the parameter values used to perform the numerical simulations. (**b**) The critical $\Gamma _{{\text {c}}}$ value, proportional to the critical current density, as a function of $h_y/h_{yc}$ for $\chi = +1$ and for several values of $\kappa$, as indicated. The red dashed line corresponds to $D=0$.

The stability domains in the $(\kappa /h_{yc},h_y/h_{yc})$ plane are represented in Fig. 2a. The shaded region is the stability domain in presence of DMI for $\chi =+1$. In this case the soliton is metastable even in a region with $\kappa >0$, where the UMA has a destabilizing effect, and the stability is provided solely by the DMI. For $\kappa >0$ the magnetic moment is aligned with the field only if $h_y>\kappa$. Hence, the magnetic texture we are considering is stable only if $h_y>\kappa$. Otherwhise, the whole system, not just the soliton, is unstable against tilting towards the $\hat{z}$ direction. The line $h_y=\kappa$, which marks the onset of this instability, is represented by the dashed blue line in Fig. 2a, and meets the upper blue curve, which signals the soliton instability at high field, at the notorious cusp. It is worthwile to mention that below the $h_y=\kappa$ line, for $\kappa >0$ and not too large, solitons with a magnetic moment along the $\hat{z}$ direction exist and are metastable, at least if $h_y$ is sufficiently large. However, these objects are beyond the scope of the present work, which concentrates in monoaxial helimagnets with easy-plane anisotroy. The green dotted line represents the stability boundary in absence of DMI for solitons of both helicities, since they are degenerate. Finally, the orange dashed line represents the stability boundary for $\chi =-1$ in presence of DMI. In both cases the UMA is necessary to provide stabilitiy, so that both lines end at $h_y=0$ for $\kappa =0$. It is evident that the DMI enlarges the stability domain of the $\chi =+1$ soliton, while it shrinks the stability domain of the $\chi =-1$ soliton. Without UMA, the $\chi = +1$ soliton is stable for $h_y\lesssim 2.15 h_{yc}$, while the $\chi =-1$ soliton is unstable for any $h_y$. For ${\text {CrNb}}_3{\text {S}}_6$, which has a large anisotropy ($\kappa \approx -5$), the stability region of the $\chi =+1$ soliton is much broader: $h_y\lesssim 6.5 h_{yc}$. Therefore, a metastable soliton can be obtained in a broad region of out-of-plane magnetic fields, $B_y$.

## Response of isolated chiral solitons to external currents

### Steady motion

We address now the question about how a chiral soliton, which can be obtained in the metastability region shown in Fig. 2a, dynamically respond to external stimuli, particularly an applied polarized current. Contrarily to domain walls, the chiral soliton does not move steadily under the application of a constant out-of-plane magnetic field. In the case of domain walls, the applied field favours the domain with magnetization aligned with it, which grows at the expenses of the other domain, and the domain wall moves steadily. In the case of the soliton, the magnetization is symmetric about its center, and the effect of the applied field is the same on both sides of the soliton. The system then reaches a new equilibrium state to minimize the Zeeman energy within the soliton, but no steady motion takes place. It is however possible to move the soliton steadily by applying a polarized electric current, with density $\vec {j}$, which delivers the spin transfer torque^52,53^

12 $$\begin{aligned} {\vec{\tau} } = -b_j( \vec {j}\cdot \nabla ){\hat{n}}+ \beta b_j {\hat{n}}\times ( \vec {j}\cdot \nabla ){\hat{n}}, \end{aligned}$$

with $b_j=P\mu _{\text {B}}/(|e|M_{\text {s}})$, where *P* is the polarization degree, *e* is the electron charge, and $\mu _B$ is the Bohr magneton. The first term is the reactive (adiabatic) torque and the second term the dissipative (non-adiabatic) torque^54^, whose strength is controlled by the nonadiabaticity coefficient $\beta$.

We take the current density ${\vec{j}} = - j \hat{z}$, and look for a steady solution of the LLG equation (2) corresponding to a soliton that moves rigidly with constant velocity, *v*, along the $\hat{z}$ direction. The general steady solution is characterized by two functions, $\theta (w)$ and $\varphi (w)$, of the variable $w=q_0(z-vt)$, with BCs: $\theta (\pm \infty ) = \pi /2$, $\varphi (-\infty )=0$ and $\varphi (+\infty ) = \chi 2\pi$. The LLG equations for steady motion can be cast into the form

13 $$\begin{aligned} \theta ^{\prime \prime }= \,& {} (\varphi ^{\prime \,2}-2\varphi ^\prime+k)\sin \theta \cos \theta -h_y\cos  \theta \cos\varphi - \Omega \theta ^\prime + \Gamma \sin \theta \varphi ^\prime , \end{aligned}$$

14 $$\begin{aligned} \sin \theta\varphi ^{\prime \prime }=\, & {} h_y\sin \varphi - 2 \, (\varphi ^\prime -1)\cos \theta \theta ^\prime - \Gamma \theta ^\prime - \Omega \sin \theta \varphi ^\prime \qquad \end{aligned}$$

where the primes stand for derivatives with respect to *w* and

15 $$\begin{aligned} \Omega = \frac{\alpha }{v_0}\Big (v-\frac{\beta }{\alpha }b_jj\Big ), \quad \Gamma = \frac{1}{v_0} \big (v - b_j j\big ). \end{aligned}$$

Notice that the spin transfer torque, the Gilbert damping, the nonadiabaticity coefficient, and the soliton velocity enter the equations of motion only through the constants $\Omega$ and $\Gamma$.

The Boundary Value Problem (BVP) defined by Eqs. (13) and (14) and the soliton BCs has no solution in general. To obtain a solution it is necessary to impose some relation between $\Omega$ and $\Gamma$, which in its turn determines a relation between the soliton velocity, *v*, and the applied current intensity, $j$. To see this, let us split the BVP into two pieces, one for $w\le 0$ and another one for $w\ge 0$, with the specified soliton BCs for $w\rightarrow \pm \infty$ supplemented with $\theta =\pi /2+\bar{\theta }_0$ and $\varphi =\pi$ at $w=0$. These two BVP have generically a solution, and have been numerically solved by a relaxation method. A solution of the complete BVP, for $-\infty<w<\infty$, is obtained from the two restricted BVP if the derivatives $\theta ^\prime$ and $\varphi ^\prime$ are continuous at $w=0$. Generically, these two conditions cannot be simultaneously satisfied by tuning the single degree of freedom at our disposal, $\bar{\theta }_0$. Hence, we have to tune $\Omega$ and $\Gamma$ to get the complete solution. It turns out that $\varphi ^\prime$ is continuous if and only if $\Omega =0$, whatever $\bar{\theta }_0$, which can be tuned to enforce the continuity of $\theta ^\prime$. Therefore, from Eq. (15) we get

16 $$\begin{aligned} v = \frac{\beta }{\alpha }b_jj, \end{aligned}$$

and in this case $\Gamma$ becomes proportional to the current intensity: $\Gamma =(\beta /\alpha -1) b_jj/v_0$. We see that the steady velocity increases linearly with the current density, with a mobility $m=(\beta /\alpha )b_j$ which is independent of the system parameters $\kappa$ and $h_y$. The same behavior occurs for domain walls^54^, for $360^\circ$ domain walls^43,44^, and for the CSL at weak field and weak current intensity^55^. Thus this seems to be a universal feature of the response of one dimensional magnetic solitons to polarized currents.

Equation (16) implies that $v=0$ if $\beta =0$, so that the steady solution is indeed static if there is no dissipative torque. In that case $\Gamma =-b_jj/v_0$ and the soliton reaches a different equilibrium state, with no motion, after applying the current. Notice also that the case $\beta =\alpha$ is special, since then $\Omega =0$ and $\Gamma =0$, and therefore Eqs. (13) and (14) are independent of the applied current. This means that in this case the soliton is rigidly dragged by the current, with velocity $v=b_jj$, without changing its static shape.

By increasing the current, $\bar{\theta }_0$ increases from its static value $\bar{\theta }_0=0$. At sufficiently large $\Gamma$ a second, *unstable*, solution of the BVP, with larger $\bar{\theta }_0$, appears. At a certain $\Gamma = \Gamma _c$, which depends on the system parameters, the stable and unstable branches meet and the steady solution becomes unstable (see Supplementary Information S1). Thus, no steady moving soliton exists above this critical current. If $\beta =\alpha$, Eqs. (13) and (14) are independent of the current, and there is no critical current. The critical current decreases with $h_y$ and increases with $\kappa$, as shown in Fig. 2b. This reflects the fact that the field tends to destabilize the soliton while DMI and UMA tend to stabilize it. The critical current vanishes when the applied field attains a value that coincides with the destabilizing field represented in Fig. 2a. This means that, as expected, unstable solitons are destroyed by infinitesimal current densities, since, as we shall see in next section, current densities higher than the critical values destroy the soliton, driving the system to the homogeneous FM state. On the other hand, it is worthwile to stress that the mobility is independent of $\chi$ if the solitons of both helicities are metastable. However, for a given current density the soliton profiles depend on $\chi$, and, as expected, the critical current is much smaller for $\chi =-1$ (see Supplementary Information S1).

A similar scenario is observed for moving domain walls^54^, in which the steady motion ceases when the current density is high enough. However, for high currents the steady motion is replaced by a precesional motion in the case of domain walls, while the chiral solitons studied here are destroyed by high currents.

In monoaxial helimagnets, the response of the CSL to a spin transfer torque delivered by a polarized current was studied by Kishine and Ovchinnikov^55^ in the weak magnetic field limit. These authors showed that the CSL reachs also a steady motion regime at low current intensities if a non-adiabatic spin transfer torque is delivered. The mobility is proportional to the current intensity, as in Eq. (16). However, the behaviour of the CSL in response to large currents was not investigated in that work. The steady motion of the CSL can be addressed in the whole magnetic field regime, including the weak limit, and for arbitrary current intensities, by solving Eqs. (13) and (14) with the appropriate BCs. As found by Kishine and Ovchinnikov, relation (16) holds also for the CSL in the weak field limit, presumable indicating that this BVP will have a solution only if $\Omega =0$, as in the isolated soliton case studied here. We also expect that a high enough current density will destroy the CSL, driving it to the homogeneous FM state. However, contrarily to the case of the isolated soliton, which is a metastable state, the CSL will reappear when the current is switched off, since it is the equilibrium state in absence of current.

### Nonsteady issues

Two important questions are not addressable by the BVP, which only describes the steady motion: (1) the fate of the soliton for $j>j_c$, and (2) whether the steady moving regime is reached by applying a current to a static soliton. To answer these questions, we performed numerical simulations of the LLG equation using the MuMax3 code^56–58^, in which we have implemented the monoaxial DMI. We used the following parameters, appropriate to ${\text {CrNb}}_3{\text {S}}_6$: $A=1.42\,{\text {pJ/m}}$, $D = 369\,\upmu {\text {J/m}}^2$, $K=-124\,{\text {kJ/m}}^3$, $M_{\text {S}}=129\,{\text {kA/m}}$, $\alpha =0.01$, $\beta =0.02$, and $P=1$. In addition, we set $B_y=300\,{\text {mT}}$ which is larger than the stability limit of the CSL $B_{y,{\text {c}}} = 230\,{\text {mT}}$. These values correspond to $q_0=0.13$ nm$^{-1}$, $\kappa =-5.17$ and $h_y=0.807$, and are indicated by the red star in Fig. 2a. The numerical solution of the BVP for this set of parameters gives $\Gamma _c = 1.2405$ (see Fig. 2b). As a test of the code, we have obtained that, in absence of applied magnetic field, the system relaxes to a helical state with wave number $q_0$, and that a metastable chiral soliton can be retained for a broad $h_y$ range.

**Figure 3:**
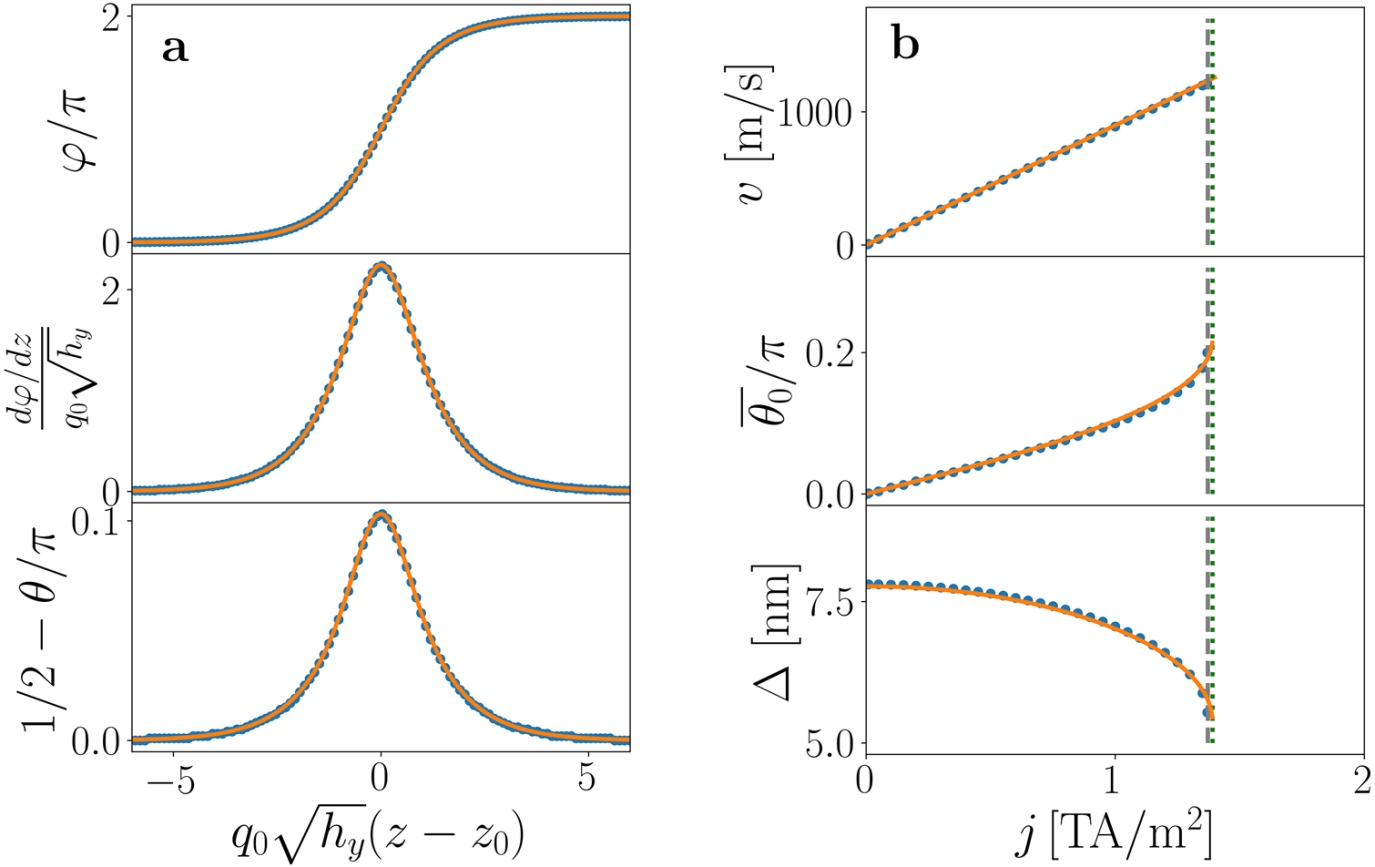
Steady motion of the chiral soliton. (**a**) Steady profiles for $\kappa =-5.17$, $h_y=0.807$, and $\Gamma =0.89$ ($j = 1\,{\text {TA/m}}^2$). Circles correspond to numerical simulations and lines to the BVP. (**b**) Velocity and soliton parameters as a function of the applied current density *j*. The steady velocity increases linearly with the current, with mobility $m = (\beta /\alpha ) b_j$, as indicated by the continuous line (top panel). Middle and bottom panels: $\bar{\theta }_0$, (tilt of the magnetization in the *z* direction) increases with *j*, whilst the soliton width $\Delta$ decreases. Both quantities show a considerable change when the critical current $j_c = 1.372\,{\text {TA/m}}^2$, indicated by the vertical dashed line, is approached. Continuous lines correspond to the solution of the corresponding BVP. Vertical dashed and dotted lines correspond to the critical values $j_c$ obtained with numerical simulations and with the BVP, respectively.

The shape of the steady moving solution for $\chi =+1$ is displayed in Fig. 3a for $j = 1\,{\text {TA/m}}^2$ ($\Gamma =0.89$). Continuous lines correspond to the solution of the BVP and circles to the steady profile found by numerical simulations of the LLG equation, showing good agreement between them. The bottom panel in Fig. 3a shows that the magnetization in the soliton is tilted towards $\hat{z}$, the direction of the velocity. Let $z_0$ be the center of the soliton, given by the maximum of $\varphi ^\prime$, where now the prime stands again for derivative respect to *z*. The tilt angle is the deviation of the polar angle from $\pi /2$ in its center, $\bar{\theta }_0 = \pi /2 - \theta (z_0)$. The soliton width $\Delta$ can be defined in terms of $\varphi '$ as $\Delta ^2 = \int (z-z_0)^2 \varphi '(z)^2dz/\int \varphi '(z)^2dz$, The values of $\Delta$ and $\bar{\theta }_0$ depend on the applied current density and on the system parameters. Figure 3b displays the steady velocity, *v*, and $\bar{\theta }_0$ and $\Delta$ as a function of *j*.

**Figure 4:**
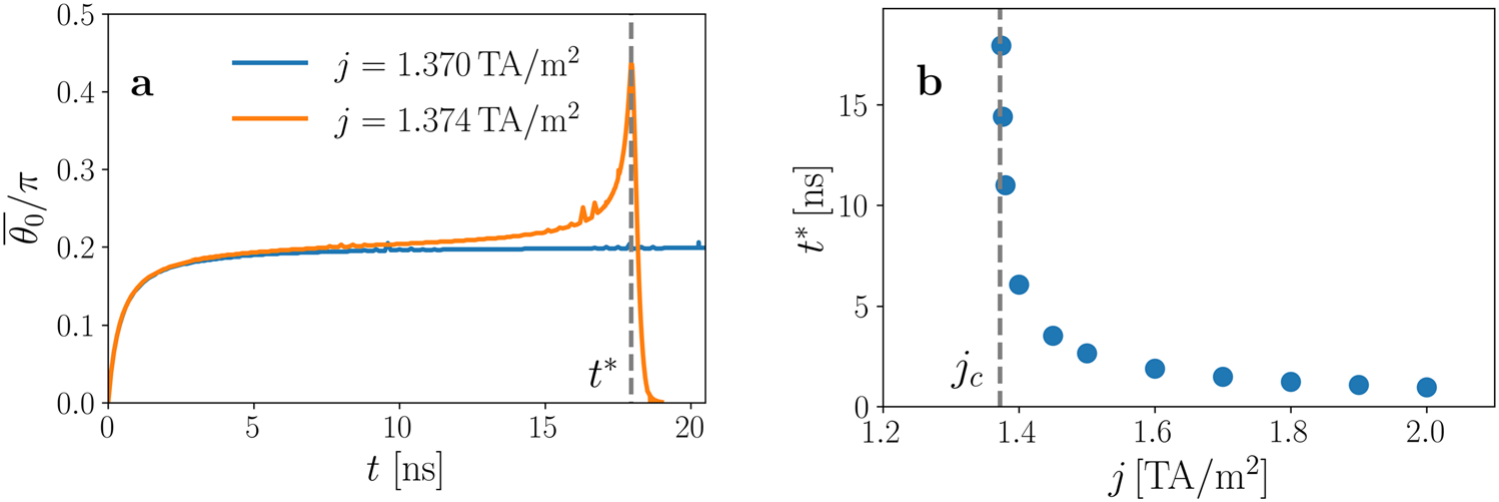
Instability of the chiral soliton under an applied current density. (**a**) Evolution with time of the tilt angle $\bar{\theta }_0$ around the critical current $j_c$. The vertical line indicates the value of $t^*$ for $j= 1.374\,{\text {TA/m}}^2$, beyond which the magnetization in the center of the soliton abruptly goes to the *y* direction. (**b**) Dependence on the current density of the instability time $t^*(j)$, showing how it seems to diverge when approaching $j_c = 1.372\,{\text {TA/m}}^2$ from above.

Numerical simulations show that the system, starting from the metastable static soliton, reaches the steady motion state if the current is below the critical current, $j_{\text {c}} = 1.372\,{\text {TA/m}}^2$, which corresponds to $\Gamma = 1.224$, in good agreement with the value of $\Gamma _c$ predicted with the BVP. Currents higher than $j_{\text {c}}$ destroy the soliton and drives the system to the FM state. Figure 4 displays results of numerical simulations that clarify the fate of the soliton upon application of a supercritical current. Figure 4a presents the temporal evolution of $\bar{\theta }_0$ for $j=1.370\,{\text {TA/m}}^2$, for which a steady soliton motion is reached, and for $j=1.374\,{\text {TA/m}}^2$, where no steady solitonic state is attained at long times. The dotted vertical line indicates the time $t^* = 17.95$ ns when the soliton is destroyed, which is anticipated by the sudden increase of $\bar{\theta }_0$. The dependence of $t^*$ on the value of the supercritical current density is presented in Fig. 4b, showing how it tends to diverge when reaching $j_c$ from above. Beyond $t^*$, the system goes to a FM state with the magnetization completely oriented along the direction of the external field.

The critical current resembles the one appearing in domain walls^54^ and is tantamount to the Walker breakdown field^59–61^. However, currents beyond the Walker breakdown do not destroy the domain wall, but induce a non-steady precesional motion. This is a major difference between the chiral soliton and domain wall steady motion.

## Discussion

We have shown that single chiral solitons can be metastably retained in monoaxial helimagnets. The metastability of the soliton is guaranteed by the Dzyaloshinskii–Moriya interaction and/or the uniaxial magnetic anisotropy. For a given material, the limit of metastability is controlled externally by the applied magnetic field. For large magnetic anisotropy energy the stability region is significantly broad, reaching magnetic field values more than six times higher than the critical value at which the CSL ceases to be stable. This opens the possibility to control the chiral soliton in a broad field range.

We have also shown that the application of an external polarized electric current serves to effectively control the chiral soliton, which moves steadily with a velocity proportional to the current density, and with the mobility given by the ratio between the nonadiabaticity and the Gilbert damping coefficients. Notably, the soliton is destabilized, and destroyed, when the current density exceeds a critical value which is controlled by the applied magnetic field. The destruction of the soliton by supercritical currents can be a very useful tool to manipulate information in potential spintronic devices that use the presence or absence of solitons as bits. Other types of magnetic solitons, namely domain walls and $360^{\circ }$ domain walls, show a similar behaviour. However, a distinguishing and very interesting feature of the chiral solitons studied here is that the metastability, including the critical current density, is controlled by the applied magnetic field. In summary, the controlled motion of chiral solitons presented here opens a new route to the development of spintronic devices based in topological structures.

## Methods

### Stability of the static soliton and steady motion solution

The lowest lying eigenvalue of the operator $K_t$ is analytically known^62^ since $K_t$ corresponds to a Pöschl–Teller potential. Without DMI, the $K_z$ operator corresponds also to a Pöschl–Teller potential and its spectrum is analytically known. In presence of DMI, the lowest lying eigenvalue of $K_z$ is not analytically known and we computed it numerically, by discretizing the operator on a one dimensional finite grid in the simplest way and using the software routines ARPACK^63^ to get its eigenvalues. We repeat the computations increasing the number of grid points and decreasing the grid spacing to control the finite volume and discretization effects.

The BVP that determines the steady motion has been solved numerically by discretizing the nonlinear equations (13) and (14) in a finite one dimensional grid and using a relaxation method to obtain the solution^64^. Again, the computations were performed with several values of the number of grid points and the grid spacing, to control the finite volume and discretization effects.

### Micromagnetic simulations

Numerical simulations of the magnetization dynamics were performed using the MuMax3 computational code^56–58^. The current release of the code includes implementations of the DMI in its bulk and interfacial form. Recently, so-called micromagnetic standard problems for ferromagnetic materials with DMI interactions has been proposed by Cortés–Ortuño *et al* in Ref.^65^. Closely following the implementation of DMI interaction for crytals within the $D_{2d}$ symmetry class in^66^, we have implemented the uniaxial DMI. Notice that the coordinate system (*x*, *y*, *z*) we are using, shown in Fig. 1 in the main text, is equivalent to (*y*, *z*, *x*) in the MuMax3 implementation. In the present notation, the $D_{2d}$ DMI energy density term implemented in^65^ has the form

17 $$\begin{aligned} e_{{\text {DMI}}} = D \hat{n}\cdot \left( \partial _z \hat{n}\times \hat{z}+ \partial _x \hat{n}\times \hat{x}\right) = D \left( n_x \partial _z n_y - n_y \partial _z n_x + n_y \partial _x n_z - n_z \partial _x n_y \right) . \end{aligned}$$

For uniaxial helimagnets the DMI energy density can be expressed as

18 $$\begin{aligned} e_{{\text {DMI}}} = -D \hat{z}\cdot \left( \hat{n}\times \partial _z \hat{n}\right) = -D \left( n_x \partial _z n_y - n_y \partial _z n_x \right) . \end{aligned}$$

Here the DMI vector points in the $+z$ direction, favoring helical magnetization textures rotating counter-clockwise in the $x-y$ plane. Accordingly, effective fields for the $D_{2d}$ DMI and uniaxial DMI in a two-dimensional system are

19 $$\begin{aligned} \vec{B}_{{\text {DMI}}} = \frac{2D}{M_{\text {S}}} \left[ \partial _z n_y {\hat{x}}+ \left( \partial _x n_z - \partial _z n_x \right) {\hat{y}}- \partial _x n_y {\hat{z}}\right] , \end{aligned}$$

and

20 $$\begin{aligned} \vec{B}_{{\text {DMI}}} = \frac{-2D}{M_{\text {S}}} \left( {\partial _z} {n_y} {\hat{x}}- {\partial _z} {n_x} {\hat{y}}\right) , \end{aligned}$$

respectively. Therefore uniaxial DMI corresponds to the first term (in vectorial notation) of the $D_{2d}$ DMI with opposite sign. We have thus used the implementation of Cortés–Ortuño^66^, which is based on the effective fields, without the terms associated to the second term of the energy density in vectorial notation.

The numerical simulations presented in this work have been performed without considering the demagnetizing field (see “Discussion” above) and with periodic boundary conditions in all directions, since we ignore the boundary effects in the *x* and *y* directions and consider a long system in the *z* direction. A cell size of 1 nm $\times$ 1 nm $\times$ 1 nm was used in a system with dimensions $L_x = 2\,{\text {nm}}$, $L_y= 1\,{\text {nm}}$ and $L_z = 480\,{\text {nm}}$.

### Material parameters for ${\text {CrNb}}_3{\text {S}}_6$

We have chosen materials parameters for ${\text {CrNb}}_3{\text {S}}_6$ based on the following criteria. At low temperature and low magnetic field the CSL is the stable magnetization state. The period of the CSL is $L_0 = 48\,{\text {nm}}$, so that $q_0 = 2\pi /L_0 = 0.13\,{\text {nm}}^{-1}$ is the wave number^4,67^. In terms of micromagnetic parameters we have $q_0=D/2A$.

When an out-of-plane magnetic field $B_y$ is applied the chiral soliton lattice is destabilized at the perpendicular critical field $B_{yc} = 230\,{\text {mT}}$ and the system goes to a uniform magnetization state in the out-of-plane direction^4^. Instead, if a magnetic field is applied in the *z* direction, a conical state is formed below $B_{zc} \approx 10 B_{yc} = 2.3\,{\text {T}}$, which is the parallel critical field^24^. Theoretically, when measuring magnetic fields in units of $B_0=D^2/(2AM_{{\text {S}}})$, the perpendicular and parallel critical fields are $h_{yc} = \pi ^2/16$ and $h_{zc} = 1-\kappa$, with $\kappa =4AK/D^2$. Therefore, $B_0 = B_{yc}/h_{yc} = 370\,{\text {mT}}$ and $h_{zc}/h_{yc} \approx 10$ for ${\text {CrNb}}_3{\text {S}}_6$ implies

21 $$\begin{aligned} \kappa \approx 1-\frac{10 \pi ^2}{16} = -5.17. \end{aligned}$$

Finally, the saturation magnetization can be obtained as

22 $$\begin{aligned} M_{{\text {S}}} = \frac{g \mu _B S}{a^3} = 129\,{\text {kA/m}}, \end{aligned}$$

where $g=2$ is the Landé factor, $\mu _B$ the Bohr magneton, $S=3/2$ is the spin modulus and $a=0.6\,{\text {nm}}$ is the lattice constant.

The micromagnetic parameters *A*, *K* and *D* can thus be obtained using $q_0 = 0.13\,{\text {nm}}^{-1}$, $\kappa = -5.17$ and $B_0 = 307\,{\text {mT}}$ through

23 $$\begin{aligned} A = \frac{B_0 M_{{\text {S}}}}{2 q_0^2} = 1.42\,{\text {pJ/m}}, \quad K = \frac{\kappa B_0 M_{{\text {S}}}}{2} = -124\,{\text {kJ/m}}^3, \quad D = \frac{B_0 M_{{\text {S}}}}{q_0} = 369\,\upmu {\text {J/m}}^2. \end{aligned}$$

In addition we assumed $\alpha = 0.01$ for the Gilbert damping parameter, $\beta = 0.02$ for the nonadiabaticity coefficient, and $P = 1$ for the polarization degree.

## Supplementary information

Supplementary Information.
