# Author Correction: Dynamics of chiral solitons driven by polarized currents in monoaxial helimagnets

**DOI:** 10.1038/s41598-022-06147-1

**Published:** 2022-02-08

**Authors:** Victor Laliena, Sebastian Bustingorry, Javier Campo

**Affiliations:** 1 Aragon Nanoscience and Materials Institute (CSIC‑University of Zaragoza) and Condensed Matter Physics Department, University of Zaragoza, C/Pedro Cerbuna 12, 50009 Zaragoza, Spain; 2 Instituto de Nanociencia y Nanotecnología, CNEA-CONICET, Centro Atómico Bariloche, R8402AGP Bariloche, Río Negro Argentina

Correction to: *Scientific Reports* 10.1038/s41598-020-76903-8, published online 24 November 2020

The original version of this Article contained errors in Equations 13 and 14.

Equation 13

$${\theta }^{\prime\prime}= ({\varphi }^{{{\prime}}2}-2{\varphi }^{{{\prime}}}-{h}_{y}cos\varphi )cos\theta +\kappa sin\theta cos\theta -\Omega {\theta }^{{{\prime}}}+\Gamma sin\theta {\varphi }^{{{\prime}}},$$

now reads:

$${\theta }^{\prime\prime}= \left({\varphi }^{{{\prime}}2}-2{\varphi }^{{{\prime}}}+\kappa \right)sin\theta cos\theta -{h}_{y}\mathrm{cos}\theta cos\varphi -\Omega {\theta }^{{{\prime}}}+\Gamma \mathrm{sin}\theta {\varphi }^{{{\prime}}},$$

Equation 14

$${\varphi }^{\prime\prime}= {h}_{y}sin\varphi -({\varphi }^{{{\prime}}}-2)cos\theta {\theta }^{{{\prime}}}-\Gamma {\theta }^{{{\prime}}}-\Omega sin\theta {\varphi }^{{{\prime}}}$$

now reads:

$${sin\theta \varphi }^{\prime\prime}= {h}_{y}sin\varphi -2({\varphi }^{{{\prime}}}-1)cos\theta {\theta }^{{{\prime}}}-\Gamma {\theta }^{{{\prime}}}-\Omega sin\theta {\varphi }^{{{\prime}}}$$

The original version of this Article also contained errors in Figure 2 where the graph was incorrect in panel (b), and in Figure 3 where the graphs were incorrect in panels (a) and (b). The original Figures 2 and 3 and accompanying legends appear below.

**Figure 2:**
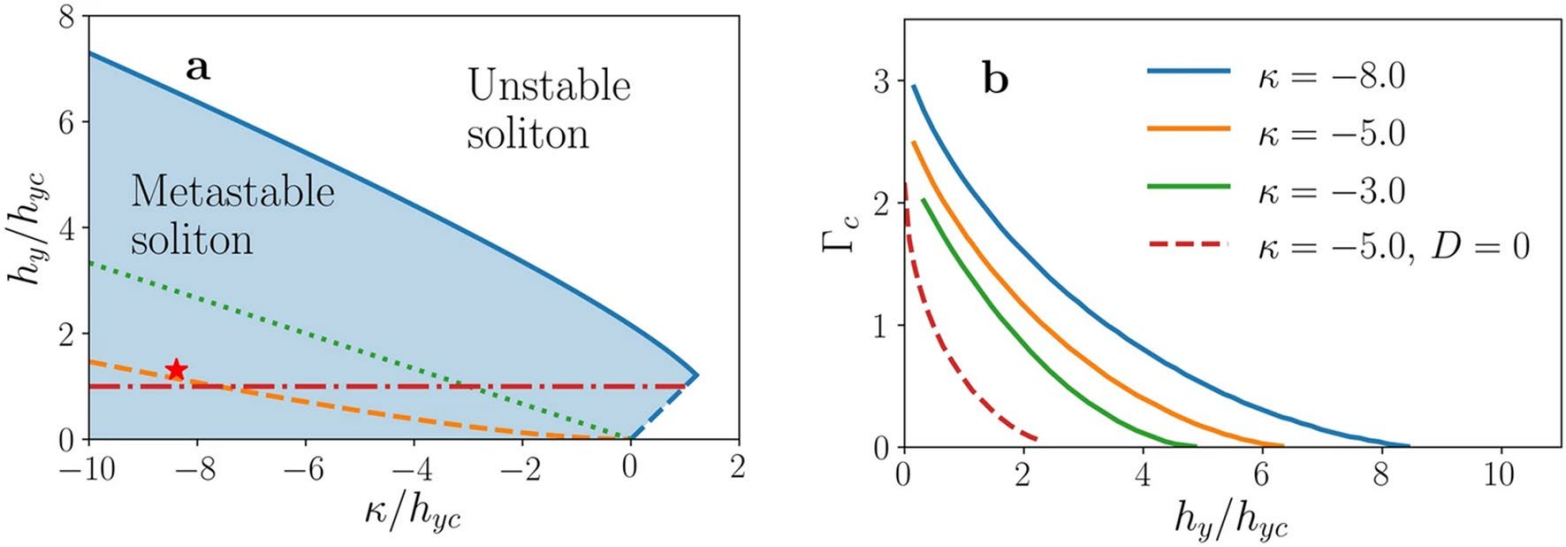
Limits of stability of the chiral soliton. (**a**) Stability diagram of the chiral soliton for *D*  >  0, as a function of anisotropy and applied field. The blue continuous line corresponds to the stability limit for $\chi = + 1$, with the dashed blue line indicating the onset of instability of the whole system against tilting towards the $\hat{z}$ direction. The orange dashed line corresponds to the stability limit for $\chi = - 1$ case. The green dotted line is the stability limit for *D* = 0 and$\chi = \pm 1$. Below the red dash-dotted line the FM state is itself metastable, the ground state being a CSL. The red star indicates the parameter values used to perform the numerical simulations. (**b**) The critical Γ_c_ value, proportional to the critical current density, as a function of ${{h}}_{{{y}}} /{{h}}_{{{{yc}}}}$ for $\chi = + 1$ and for several values of $\kappa$ , as indicated. The red dashed line corresponds to *D* = 0.

**Figure 3:**
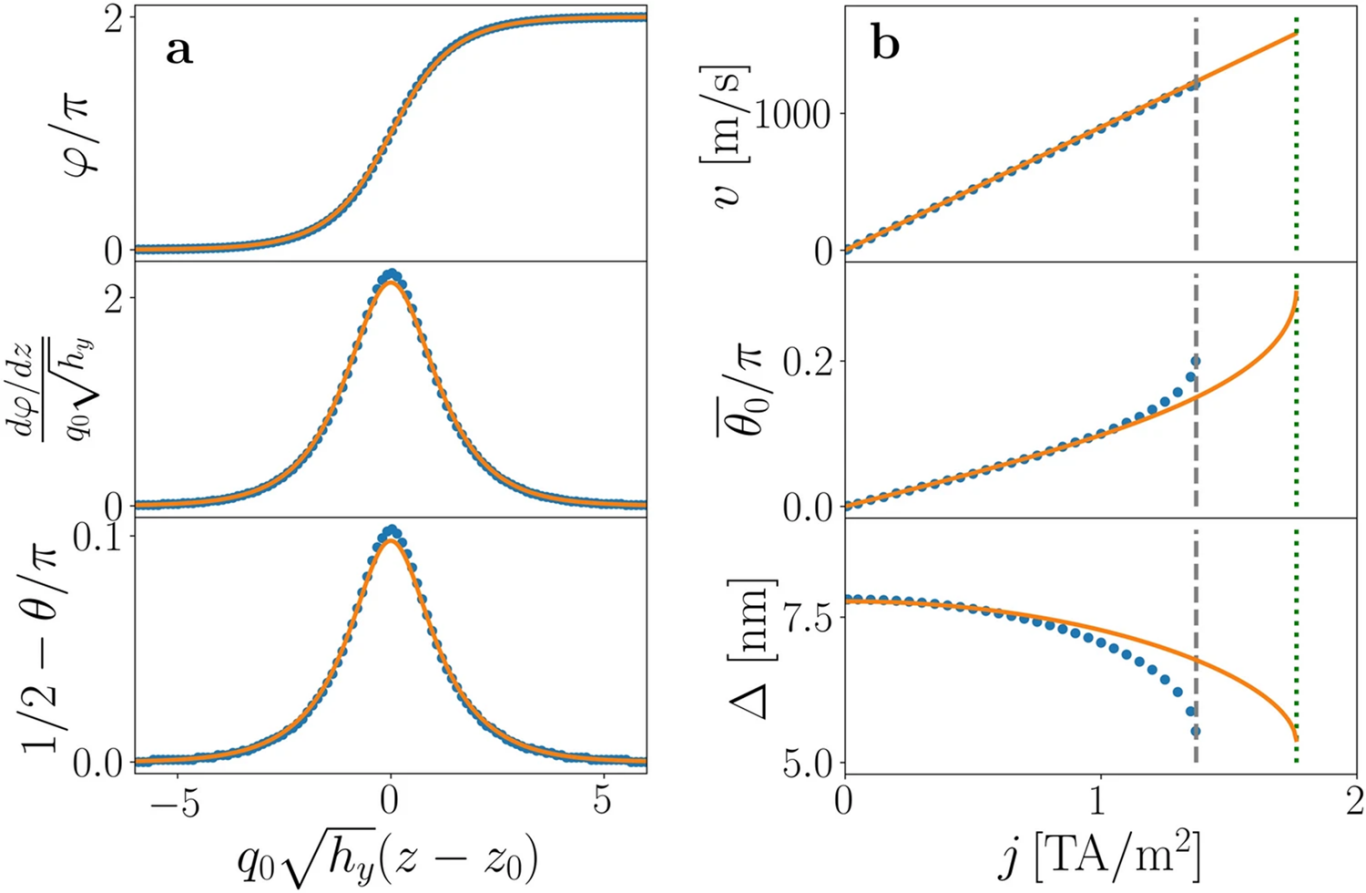
Steady motion of the chiral soliton. (**a**) Steady profiles for $\kappa = - 5.17,h_{y} = 0.807$, and $\Gamma = 0.89\,\,(j = 1\,\,\, {\text{TA/m}}^{2} )$. Circles correspond to numerical simulations and lines to the BVP. (**b**) Velocity and soliton parameters as a function of the applied current density *j*. The steady velocity increases linearly with the current, with mobility $m = (\beta /\alpha )b_{j}$, as indicated by the continuous line (top panel). Middle and bottom panels: $\overline{\theta }_{0}$, (tilt of the magnetization in the *z* direction) increases with *j*, whilst the soliton width $\Delta$ decreases. Both quantities show a considerable change when the critical current $j_{c} = 1.372\, \,\,{\text{TA/m}}^{2} ,$ indicated by the vertical dashed line, is approached. Continuous lines correspond to the solution of the corresponding BVP. Vertical dashed and dotted lines correspond to the critical values $j_{c}$ obtained with numerical simulations and with the BVP, respectively.

In addition, in the Response of isolated chiral solitons to external currents section, under the subheading ‘Nonsteady issues’,

“The numerical solution of the BVP for this set of parameters gives $\Gamma_{c} = 1.5735$ (see Fig. 2b).”

now reads:

“The numerical solution of the BVP for this set of parameters gives $\Gamma_{c} = 1.2405$ (see Fig. 2b).”

“Numerical simulations show that the system, starting from the metastable static soliton, reaches the steady motion state if the current is below the critical current, $j_{{\text{c}}} = 1.372\, \,\,{\text{TA/m}}^{2}$, which corresponds to $\Gamma = 1.224$, slightly smaller than the value of $\Gamma_{c}$ predicted with the BVP.”

now reads:

“Numerical simulations show that the system, starting from the metastable static soliton, reaches the steady motion state if the current is below the critical current, $j_{{\text{c}}} = 1.372\,\,\, {\text{TA/m}}^{2}$, which corresponds to $\Gamma = 1.224$, in good agreement with the value of $\Gamma_{c}$ predicted with the BVP.”

The original Article has been corrected.

