# Supplementary material for: Dynamics of chiral solitons driven by polarized currents in monoaxial helimagnets
Source: Sci Rep. 2020 Nov 24;10:20430. doi: 10.1038/s41598-020-76903-8 (PMC7686507; doi:10.1038/s41598-020-76903-8)
Supplement: Supplementary file 1 — Supplementary Information. [file 41598_2020_76903_MOESM1_ESM.pdf]

Supplemental material to "Dynamics of chiral solitons driven by polarized currents in monoaxial helimagnets"

Victor Laliena,[1,*] Sebastian Bustingorry,[2,†] and Javier Campo[1,‡]

[1] *Aragon Material Science Institut (CSIC – University of Zaragoza) and Condensed Physics Matter Department, University of Zaragoza C/ Pedro Cerbuna 12, 50009 Zaragoza, Spain*

[2] *Instituto de Nanociencia y Nanotecnología, CNEA-CONICET, Centro Atómico Bariloche, (R8402AGP), S. C. de Bariloche, Río Negro, Argentina*

(Dated: August 11, 2020)

The supplemental material contais details about computations of the chiral soliton stability domain, of the BVP that provides the steady solution, of the implementation of monoaxial DMI in the MuMax program and numerical simulations, and of the dynamics of the unfavored $\chi = -1$ chiral soliton.

S1. CHIRAL SOLITON STABILITY

The chiral soliton is metastable if the differential operators K_t and K_z defined by Eqs. (8) and (9) in the main text are (semi)positive definite. Let us write them here again for the reader convenience:

$$K_t = -\nabla^2 - \frac{1}{2}\varphi_0'^2 + q_0^2 h_y, \tag{1}$$

$$K_z = -\nabla^2 - \frac{3}{2}\varphi_0'^2 + q_0\varphi_0' + q_0^2(h_y - \kappa), \tag{2}$$

where the prime stands for derivative respect to z. By Fourier transform in x and y, and changing the variable to $w = \chi\sqrt{h_y}q_0 z$, the eigenvalues of K_t and K_z, denoted by μ_t and μ_z respectively, can be written as

$$\mu_t = q_0^2 h_y(\lambda_t + 1) + k_x^2 + k_y^2 \tag{3}$$

$$\mu_z = q_0^2 h_y(\lambda_z + 1) - q_0^2\kappa + k_x^2 + k_y^2 \tag{4}$$

where k_x and k_y are the components of the Fourier wave vector, and λ_t and λ_z are the eigenvalues of

$$\tilde{K}_t = -\frac{d^2}{dw^2} - \frac{2}{\cosh^2 w}, \tag{5}$$

$$\tilde{K}_z = -\frac{d^2}{dw^2} - \frac{6}{\cosh^2 w} + \frac{\chi}{\sqrt{h_y}}\frac{4}{\cosh w}, \tag{6}$$

respectively. From now on the prime stands for the derivative with respect to w.

Let us consider first $\tilde{K}_t$, which is the Schrödinger operator with a Pöschl-Teller potential. Its lowest lying eigenvalue is $\lambda_t = -1$ [1]. Hence, $\mu_t \geq 0$ and K_t is always semidefinite positive. This result can be quickly obtained by noticing that φ_0' is an eigenstate of $\tilde{K}_t$ with zero eigenvalue. This can be checked either by direct application of $\tilde{K}_t$ to φ', and it is due to the fact that φ' is the generator of infinitesimal translations of the soliton along z:

$$\hat{n}_0(w + \delta w) = \hat{n}_0(w) + \delta w \varphi_0' \hat{z} \times \hat{n}_0(w), \tag{7}$$

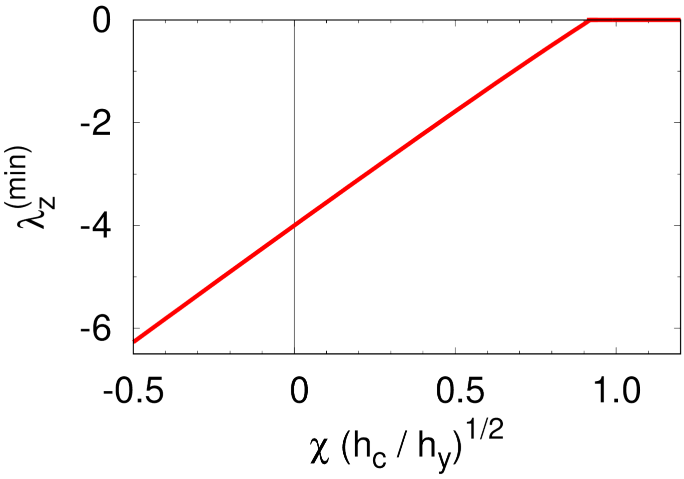

FIG. 1: Lowest lying eigenvalue of $\tilde{K}_z$.

and $\hat{n}_0(w + \delta w)$ and $\hat{n}_0(w)$ have the same energy. Given that φ_0' has no nodes (it does not vanish at any point), Sturm theorem [2] guarantees that it corresponds to the lowest lying eigenvalue.

Hence, the stability of the soliton is solely determined by the lowest lying eigenvalue of $\tilde{K}_z$, denoted by $\lambda_z^{(\mathrm{min})}$. For given χ and h_y, we have $\mu_z \geq 0$ if and only if $\kappa \leq \kappa_c$, with

$$\kappa_c = h_y\big(\lambda_z^{(\mathrm{min})} + 1\big). \tag{8}$$

Notice that the spectrum of $\tilde{K}_z$ depends only on $\chi/\sqrt{h_y}$, or, equivalently, on $\chi\sqrt{h_c/h_y}$, where $h_c = \pi^2/16$ is the critical field for soliton proliferation. If we set $\chi = 0$ in $\tilde{K}_z$, it becomes a Pöschl-Teller potential whose lowest lying eigenvalue is $\lambda_z^{(\mathrm{min})} = -4$. Hence, in absence of DMI, the chiral soliton is stable for $h_y < -\kappa/3$, and, in a simple ferromagnet, without DMI and anisotropy, it is always unstable, since $h_y \geq 0$ by definition. The lowest lying eigenvalue of $\tilde{K}_z$ as a function of $\chi\sqrt{h_c/h_y}$ is displayed in Fig. 1. The soliton (metas)tability boundary as a function of χ, κ, and h_y can be determined from Eq. (8) and Fig. 1.

S2. SOLUTION OF THE BOUNDARY VALUE PROBLEM

The soliton steady motion driven by a polarized torque is determined by the Boundary Value Problem (BVP) set by equations (12) and (13) of the main text, which we reproduce

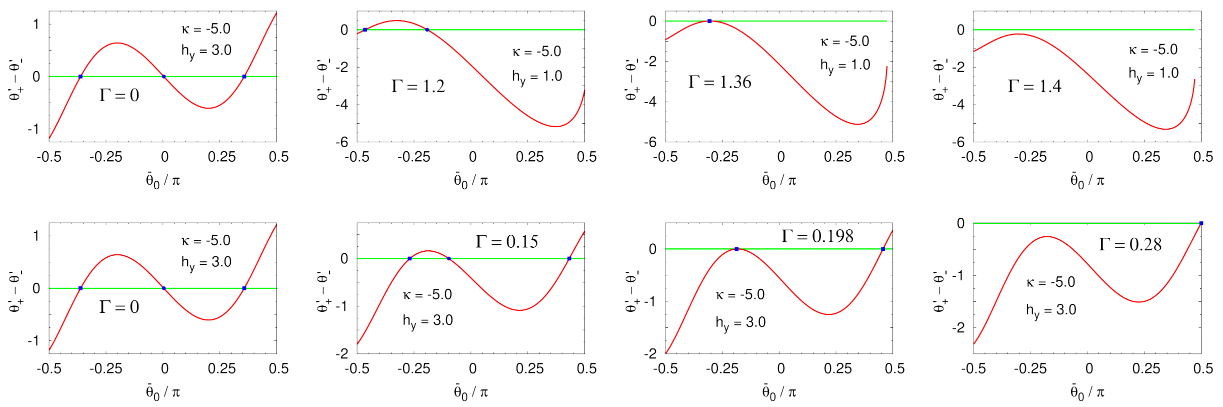

FIG. 2: Matching at $w = 0$ of the BVPs for $w \leq 0$ and $w \geq 0$, given by $\theta'_+ - \theta'_-$, as a function of $\bar{\theta}_0$, for $\Omega = 0$, several values of Γ, $\kappa = -5.0$ and $h_y = 1.0$ (top panels) and $h_y = 3.0$ (bottom panels). The blue filled circles and squares mark, respectively, stable and unstable solutions of the BVP for $-\infty < w < \infty$. The green lines show $\lim_{w\to 0^+} \varphi'(w) - \lim_{w\to 0^-} \varphi'(w)$, which is zero to machine precision.

below for the reader convenience,

$$\theta'' = (\varphi'^2 - 2\varphi' - h_y \cos\varphi)\cos\theta + \kappa \sin\theta\cos\theta - \Omega\theta' + \Gamma\sin\theta\varphi', \tag{9}$$

$$\varphi'' = h_y \sin\varphi - (\varphi' - 2)\cos\theta\theta' - \Gamma\theta' - \Omega\sin\theta\varphi', \tag{10}$$

and the boundary conditions (BCs)

$$\theta(\pm\infty) = \pi/2, \quad \varphi(-\infty) = 0, \quad \varphi(+\infty) = \chi 2\pi, \tag{11}$$

where θ and φ are functions of the variable $w = q_0(z - vt)$ and the prime stands for the derivative with respect to w.

This BVP has no solution in general. To obtain a solution it is necessary to impose some relation between Ω and Γ. To see why the BVP has no solution in general, let us analyze the general form of the solution in the asymptotic region $w \to \pm\infty$.

In the $w \to -\infty$ region, φ' and $\bar{\theta} = \pi/2 - \theta$ are exponentially small and the linearized equations have the form

$$\bar{\theta}'' = (h_y - \kappa)\bar{\theta} - \Omega\bar{\theta}' - \Gamma\varphi', \tag{12}$$

$$\varphi'' = h_y\varphi + \Gamma\bar{\theta}' - \Omega\varphi'. \tag{13}$$

The solution is $\bar{\theta} = \bar{\theta}_\nu \exp\nu z$ and $\varphi = \varphi_\nu \exp\nu z$, with ν a solution of

$$(\nu^2 + \Omega\nu - h_y + \kappa)(\nu^2 + \Omega\nu - h_y) + \Gamma^2\nu^2 = 0. \tag{14}$$

This equation has four solutions. For $\Omega = \Gamma = 0$ they are $\nu = \pm\sqrt{h_y}$ and $\nu = \pm\sqrt{h_y - \kappa}$. Hence, two values of ν are positive and two negatives. Therefore, at least for small Ω and Γ, two values of ν will have positive real part and two negative real part. Let us call ν_i, with $i = 1, \ldots, 4$, the four solutions of (14), with $i = 1, 2$ having positive real part and $i = 3, 4$ negative real part. The general asymptotic solution as $w \to -\infty$ is

$$\begin{pmatrix} \bar{\theta} \\ \varphi \end{pmatrix} = \sum_{i=1}^{4} a_i^{(-)} \mathrm{e}^{\nu_i w} \begin{pmatrix} u_i \\ v_i \end{pmatrix}. \tag{15}$$

where the vector $(u_i, v_i)^T$ is a solution of

$$\begin{pmatrix} \nu_i^2 + \Omega\nu_i - h_y + \kappa & -\Gamma\nu_i \\ \Gamma\nu_i & \nu_i^2 + \Omega\nu_i - h_y \end{pmatrix} \begin{pmatrix} u_i \\ v_i \end{pmatrix} = 0. \tag{16}$$

To satisfy the BCs for $z \to -\infty$ it is necessary (and sufficient) that $a_3^{(-)} = a_4^{(-)} = 0$.

For $w \to +\infty$ the functions $\bar{\theta} = \pi/2 - \theta$ and $\bar{\varphi} = \chi(2\pi - \varphi)$ are exponentially small and the linearized equations read

$$\bar{\theta}'' = (h_y - \kappa)\bar{\theta} - \Omega\bar{\theta}' + \chi\Gamma\bar{\varphi}', \tag{17}$$

$$\bar{\varphi}'' = h_y\bar{\varphi} - \chi\Gamma\bar{\theta}' - \Omega\bar{\varphi}'. \tag{18}$$

Notice that these equations are obtained from Eqs. (12) and (13) just replacing Γ by $-\chi\Gamma$. Hence, the general solution is

$$\begin{pmatrix} \bar{\theta} \\ \bar{\varphi} \end{pmatrix} = \sum_{i=1}^{4} a_i^{(+)} \mathrm{e}^{\nu_i w} \begin{pmatrix} u_i \\ v_i \end{pmatrix}, \tag{19}$$

where the ν_i are the solutions of (14) and the vectors $(u_i, v_i)^T$ are the corresponding solution of (16) in which Γ has to be replaced by $-\chi\Gamma$. The B.C. for $w \to +\infty$ require $a_1^{(+)} = a_2^{(+)} = 0$.

In general, a BVP can have one, many, or no solution. An Initial Value Problem (IVP), however, has one and only one solution. We may try to solve the BVP problem as an IVP with initial conditions at $w = 0$ given by

$$\varphi(0) = \pi, \quad \varphi'(0) = \varphi'_{\mathrm{ini}}, \quad \theta(0) = \theta_{\mathrm{ini}}, \quad \theta'(0) = \theta'_{\mathrm{ini}}. \tag{20}$$

The asymptotic solutions of the IVP as $w \to \pm\infty$ are given by Eqs. (15) and (19), with coefficients $a_i^{(\pm)}$ that are functions of the initial conditions φ'_{ini}, θ_{ini}, and θ'_{ini}, and of Ω and Γ. The solution of the IVP will be a solution of the BVP if

$$a_3^{(-)} = a_4^{(-)} = a_1^{(+)} = a_2^{(+)} = 0. \tag{21}$$

These four equations cannot be solved in general only with the three initial conditions as variables. They may be satisfied, however, if we tune either Ω, or Γ, or both. The initial conditions, however, suffice to enforce either $a_3^{(-)} = a_4^{(-)} = 0$ or $a_1^{(+)} = a_2^{(+)} = 0$. Therefore, the BVPs restricted either to the positive ($-\infty < w \leq 0$) or negative ($0 \leq w < +\infty$) region, with the BCs

$$\varphi(0) = \pi, \quad \theta(0) = \theta_{\text{ini}}, \quad \varphi(-\infty) = 0, \quad \varphi(+\infty) = \chi 2\pi \quad \theta(\pm\infty) = \theta_{\text{ini}}, \tag{22}$$

have a solution, that can be obtained by solving the IVP and using φ'_{ini} and θ'_{ini} to enforce the BCs on each side ($w < 0$ or $w > 0$). Of course, the values of φ'_{ini} and θ'_{ini} will be different for each of the two BVPs.

We solved the BVP by splitting it into two pieces, one for $w \leq 0$ and another one for $w \geq 0$, with the BCs of Eqs. (22). We solved them numerically, using a relaxation method. A solution of the complete BVP, for $-\infty < w < \infty$, is obtained from the two restricted BVP if the derivatives θ' and φ' are continuous at $w = 0$. To satisfy these two conditions, we have θ_{ini} at our disposal as a variable, and, given that this is not enough, we need to tune also Ω and Γ. It turns out that φ' is continuous at $w = 0$ if and only if $\Omega = 0$, whatever θ_{ini} or Γ. Therefore, we set $\Omega = 0$ and use θ_{ini} to enforce the continuity of θ' at $w = 0$. The condition $\Omega = 0$ determines the soliton velocity through Eq. (15) of the main text, which is

$$v = \frac{\beta}{\alpha} b_j j. \tag{23}$$

Then, $\Gamma = (\beta/\alpha - 1) b_j j / v_0$, which remains as a free parameter controlled by the current density.

Let us write $\theta_{\text{ini}} = \pi/2 - \bar{\theta}_0$. For $\Omega = 0$ and given Γ, we solved the two restricted BVP for a sufficiently dense mesh of $\bar{\theta}_0$ from $-\pi/2$ to $\pi/2$ (since the polar angle θ takes values between 0 and π). Defining $\theta'_{\pm} = \lim_{w \to 0^{\pm}} \theta'(w)$, we obtain a solution of the complete BVP if $\theta'_{+} - \theta'_{-} = 0$. Fig. 2 shows $\theta'_{+} - \theta'_{-}$ as a function of $\bar{\theta}_0$ for $\kappa = -5.0$ and the values of h_y and Γ displayed in the legends. When several zeros of $\theta'_{+} - \theta'_{-}$ appear, the steady solution corresponding to θ_0 closest 0 is stable and the other unstable.

Fig. 3 displays the solutions of the BVP, characterized by $\bar{\theta}_0$, as a functionof Γ in several cases. The solid red lines are the stable solutions and the broken green lines the unstable solutions. The stable solution becomes unstable at Γ_c, when it meets the unstable branch. For low h_y (top panels) no steady solution exists for $|\Gamma| > |\Gamma_c|$. For higher h_y (bottom

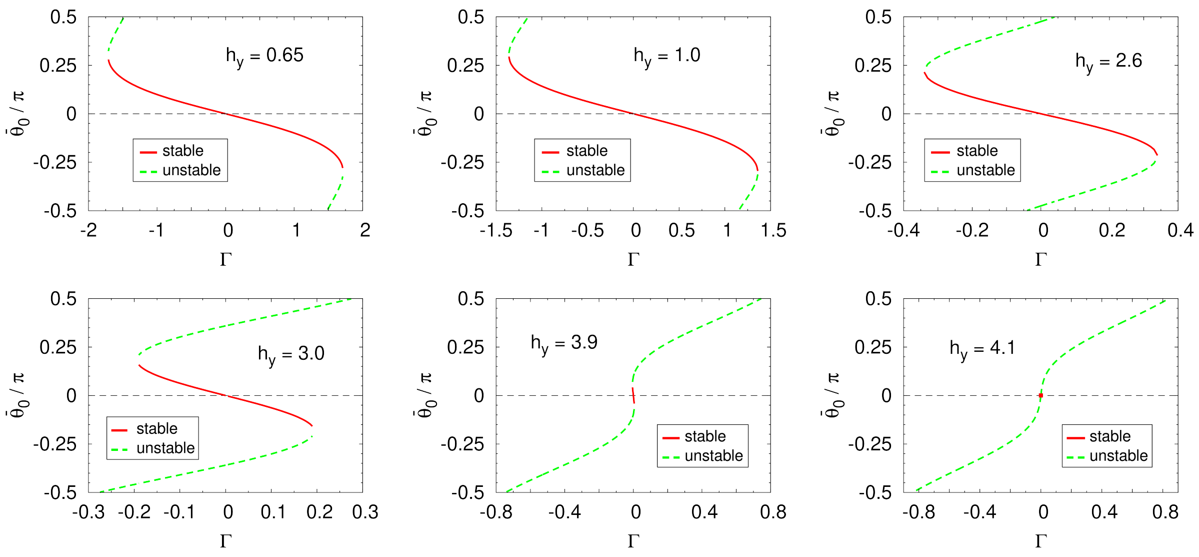

FIG. 3: Solutions of the BVP, parametrized by $\bar{\theta}_0$, as a function of Γ for $\kappa = -5.0$ and the h_y displayed in the legend. The solid red lines are the stable solutions. The broken green lines are unstable solutions. On the soliton stability boundary, $h_y \approx 4.1$, the stable solution is reduced to the point $\Gamma = 0$ and $\bar{\theta}_0 = 0$.

panels) unstable steady solutions exist for $|\Gamma| > |\Gamma_c|$. As h_y approaches from below the soliton stability boundary, which for $\kappa = -5.0$ corresponds to $h_y \approx 4.1$ [Fig. 2(a) of the main text], the stable solution branch shrinks to zero, and, evidently, for higher values of h_y even the static ($\Gamma = 0$) solution is unstable.

S3. DYNAMICS OF THE $\chi = -1$ CHIRAL SOLITON

When $D > 0$ the $\chi = +1$ soliton if favored against the one with the opposite chirality $\chi = -1$. Despite that, the $\chi = -1$ soliton can also be obtained as a metastable state of the system. Figure 2(a) of the main text shows that the region of metastability of the $\chi = -1$ soliton is reduced as compared with that of the $\chi = 1$ soliton. This means that a metastable $\chi = -1$ soliton can also be retained and forced under an applied current density. We have numerically tested this by driving the unfavored $\chi = -1$ soliton at $B_y = 0.2\,\mathrm{T}$.

Figure 4(a) shows the evolution the velocity of the chiral soliton with the current density (upper paner). The mobility is $m = (\beta/\alpha) b_j$ and does not depend on the form or sign of the soliton. The tilt angle in the z direction, $\bar{\theta}_0$, is shown in the middle panel of Fig. 4(a) while

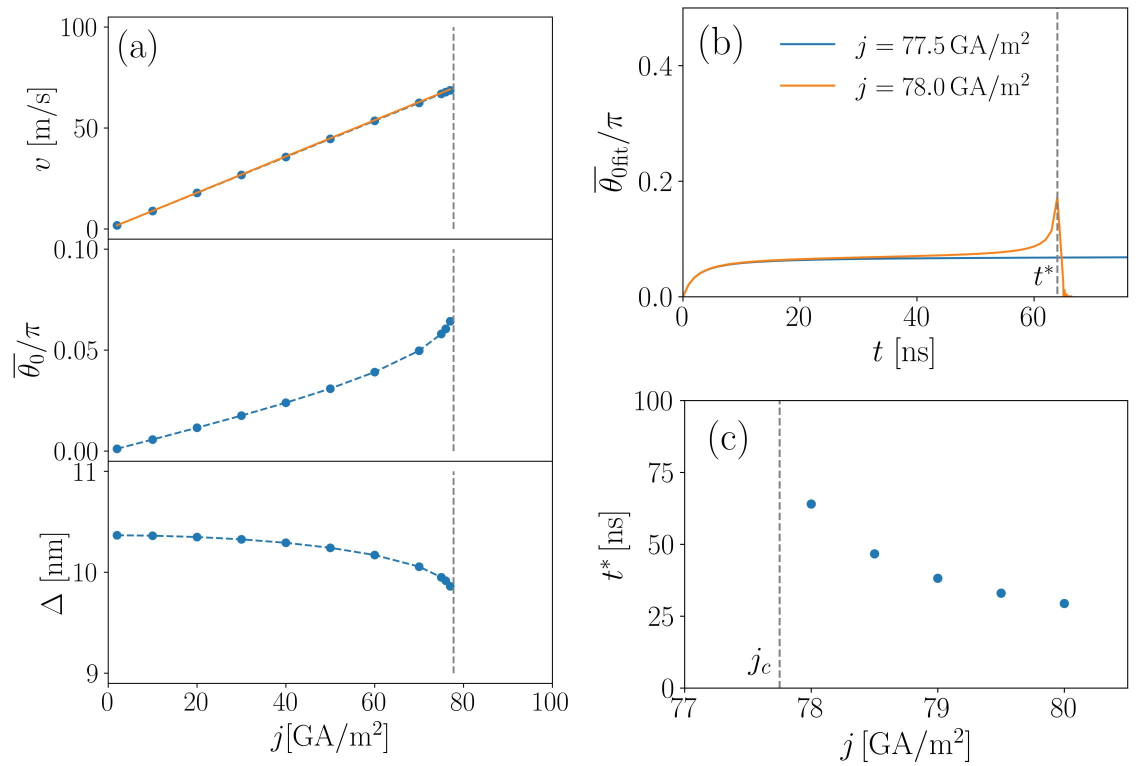

FIG. 4: (a) Velocity and soliton parameters as a function of the applied current density j for the unfavored $\chi = -1$ soliton. The steady velocity increases linearly with the current, with the same mobility $m = (\beta/\alpha)b_j$ as for the $\chi = +1$ soliton, as indicated by the continuous line (top panel). The tilt of the magnetization in the z direction $\bar{\theta}_0$ is shown in the middle panel, presenting a noticeable increase when reaching $j_c = 77.75\,\mathrm{GA/m^2}$ (vertical lines). Soliton width Δ decreases with the current density, as shown in the bottom panel. (b) Evolution with time of the tilt angle $\bar{\theta}_0$ around the critical current j_c. The vertical line indicates the value of t^* for $j = 78.0\,\mathrm{GA/m^2}$, beyond which the magnetization in the center of the soliton abruptly goes to the y direction. (c) Dependence on the current density of the instability time $t^*(j)$, showing how it seems to diverge when approaching $j_c = 77.75\,\mathrm{GA/m^2}$ from above.

the soliton width Δ is presented in the bottom panel. A noticeable change is observed in both quantities when approaching the critical curren density $j_c = 77.75\,\mathrm{GA/m^2}$, indicated by vertical lines. The evolution of the tilt angle $\bar{\theta}_0$ around the critical current density is presented in Fig. 4(b), with t^* indicating the time beyond which the single soliton is destroyed. Finally, Fig. 4(c) shows how t^* increases when j_c is approached from above.

* laliena@unizar.es

† sbusting@gmail.com

‡ javier.campo@csic.es

[1] G. P. Pöschl and E. Teller, Z. Phys. **83**, 143 (1933).

[2] N. Dunford and J. T. Schwarz, *Linear Operators* (Wiley, New York, 1964).
